# Interplay of the Influence of Crosslinker Content and Model Drugs on the Phase Transition of Thermoresponsive PNiPAM-BIS Microgels

**DOI:** 10.3390/gels8090571

**Published:** 2022-09-08

**Authors:** Daniel Schlattmann, Monika Schönhoff

**Affiliations:** Institute of Physical Chemistry, University of Münster, Corrensstr. 28-30, 48149 Münster, Germany

**Keywords:** poly(*N*-isopropylacrylamide), VPTT, crosslinker, thermoresponsive, microgel, additive, phase transition, NMR, DSC, DLS

## Abstract

The phase transition behavior of differently crosslinked poly(*N*-isopropylacrylamide)/*N*,*N*’-methylenebisacrylamide (PNiPAM/BIS) microgels with varying crosslinker content is investigated in presence of aromatic additives. The influence of *meta*-hydroxybenzaldehyde (*m*-HBA) and 2,4-dihydroxybenzaldehyde (2,4-DHBA), chosen as model drugs, on the volume phase transition temperature (VPTT) is analyzed by dynamic light scattering (DLS), differential scanning calorimetry (DSC), and ^1^H-NMR, monitoring and comparing the structural, calorimetric, and dynamic phase transition, respectively. Generally, the VPTT is found to increase with crosslinker content, accompanied by a drastic decrease of transition enthalpy. The presence of an additive generally decreases the VPTT, but with distinct differences concerning the crosslinker content. While the structural transition is most affected at lowest crosslinker content, the calorimetric and dynamic transitions are most affected for an intermediate crosslinker content. Additive uptake of the collapsed gel is largest for low crosslinked microgels and in case of large additive-induced temperature shifts. Furthermore, as temperature is successively raised, ^1^H NMR data, aided by spin relaxation rates, reveal an interesting uptake behavior, as the microgels act in a sponge-like fashion including a large initial uptake and a squeeze-out phase above VPTT.

## 1. Introduction

Within the last decades, Poly(*N*-isopropylacrylamide) (PNiPAM) is one of the most intensely studied water-soluble thermoresponsive polymers [1,2]. First investigations of the PNiPAM homopolymer and the observation of its lower critical solution temperature (LCST) were published in the late 1960s [3], followed by first reports of its corresponding microgel, crosslinked by *N*,*N*′-methylenebisacrylamide (BIS) in 1986 [4]. Microgels (µ-PNiPAM) are colloidal spherical particles, consisting of a three-dimensional chemically crosslinked network structure. Those particles exhibit a temperature-dependent swelling behavior in aqueous solvent. Below their so-called volume phase transition temperature (VPTT), microgels are highly swollen by the solvent, while above the VPTT, typically around 32 °C, a collapse of the network and phase separation occurs. As the phase transition takes place in the temperature range of the human body, these polymers are suggested for various applications such as drug delivery [5,6,7,8], tissue engineering [9,10], self-healing coatings [11], carrier for catalysts [12], or even ion-sensitive sensors [13,14]. Many physical and chemical stimuli are available [15] to influence the phase transition behavior such as salts [16], surfactants [17], solvents [18], ultrasound [19], electromagnetic radiation [20], and additives [21,22,23].

The most commonly targeted disease by gels of PNiPAM and its copolymers is cancer in its manifold forms, such as breast cancer [24], liver tumors [25,26] and other malignant tumors, which can be treated by paclitaxel [27]. The goal is to improve the deliverable drug content and optimize the therapeutic efficacy for specific drug release, supported by PNiPAM. PNiPAM nanoparticles are also considered for targeted and enhanced nose-to-brain delivery of curcuminoids to cope with consequences of a stroke, as those drugs are reported to play a potential role in relieving of cerebral ischemia [28,29]. Another approach employs PNiPAM as an adhesive for intraocular use in ophthalmology [30].

In general, PNiPAM microgels are amphiphilic, as the polymer consist of a hydrophobic backbone and isopropyl groups, while the amides in the side groups (as well as those introduced by the crosslinker BIS) are hydrophilic. [31] Hydrogen bonding between H_2_O and the polymer is responsible for its enthalpy-driven phase transition. Below the VPTT, water molecules form a “cage-like” solvent structure around moieties of the polymer. When temperature is raised to exceed the VPTT, these hydrogen bonds become unstable, resulting in a partial dehydration of the polymer and an entropically driven release of water [32]. Intramolecular hydrophobic polymer–polymer interactions are now dominant. Although this reversible process has been studied intensely [2,33,34,35], the kinetics of the phase transition of PNiPAM microgels is still under investigation and a matter of debate.

It is known that even small concentrations of additional solutes can have an influence on the LCST of linear PNiPAM chains and the VPTT of crosslinked PNiPAM/BIS microgels. A depression of the transition temperature occurs upon salt addition, and its magnitude is related to the Hofmeister series and linearly dependent on salt concentration [16]. It is also reported that the addition of cosolvents like 1-propanol to aqueous PNiPAM solutions can reduce the phase transition temperature, while it can also cause the effect of co-nonsolvency [36]. An increase in transition temperatures is observed for the addition of sodium dodecylsulfate (SDS) as a surfactant [37], whereas similar structures of sodium *n*-alkyl sulfates with intermediate chain lengths (n=5−10) can depress or increase the transition temperature in dependence of applied concentrations [38].

The application of microgels for drug delivery purposes requires fundamental knowledge about interactions between polymeric structures and model drugs [39,40]. As medically relevant drugs often exhibit complex molecular structures, small aromatic model drugs are preferentially used to mimic the former [21,23]. Such aromatic additives shift the hydrophobic coil-to-globule transition of PNiPAM and poly(*N*,*N*-diethylacrylamide) (PDEAM) homopolymers to lower temperatures [41]. Concerning PNiPAM homopolymers and microgels, detailed studies involved phenol [23,32,40], hydroxybenzenes [41], benzaldehydes [21,41], and dopamine [23,40], as well as indol [40] and its dervatives [23]. For example, an indole ring as a component of various biologically active natural products was shown to interact with PNiPAM only above the LCST [23], while all aromatic additives reduce the phase transition temperature.

Concerning the effect of the crosslinker BIS, it was accepted in literature for a long time that its influence on the VPTT is only marginal, as microgels containing different amounts of BIS showed no evidence of systematic temperature shifts [42,43,44,45,46]. Mostly, the crosslinker influence was described as a broadening of the phase transition, which stays centered at around 32 °C [42,43,44,45,46]. Published values for VPTTs from Karg et al. for µ-PNiPAM/x-BIS (x = 2, 5, 15 mol%) microgels vary in a range of 31.8–33.7 °C, but do not reflect a systematic trend, as the lowest VPTT is observed for x = 5 [44]. In 2021, the first results of systematic VPTT shifts were reported in a DLS study by Friesen et al., showing an increasing VPTT with higher crosslinker content [47].

Here, we are investigating the influence of BIS crosslinker content in combination with the effects of two model additives on the microgel phase transitions, namely *m*-Hydroxybenzene (*m*-HBA) and 2,4-Dihydroxybenzene. Swelling curves for the neat microgels and microgel-additive dispersions are measured by Dynamic Light Scattering (DLS) for a structural characterization. To account for thermodynamic insights, Differential Scanning Calorimetry (DSC) measurements are conducted. Finally, a more detailed insight into molecular dynamics and distribution of the additives is obtained by Nuclear Magnetic Resonance (NMR) spectroscopy.

The results from all methods consistently show a linear increase of the VPTT with increasing crosslinker content. An additional temperature shift Δ*T* is induced in presence of an additive. Δ*T* is negative for each crosslinker content, and the depression is more pronounced for 2,4-DHBA than for *m*-HBA. Furthermore, weakly crosslinked microgels are more sensitive to the influence of additives. This higher sensitivity is accompanied by incorporation of a larger fraction of additive upon collapse. Spin relaxation rates *R*_2_ of the additives reveal a reduction of their dynamics, which is most pronounced at low crosslinker density. Spin relaxation rates further indicate the strongest additive–polymer interactions at temperatures around the VPTT, while above VPTT additive molecules are partially “squeezed out” of the microgel.

## 2. Results and Discussion

### 2.1. Characterization of µ-PNiPAM/x-BIS µGels with Different Crosslinker Content

Our systematic synthesis approach yields µ-PNiPAM microgels containing different amounts of crosslinker BIS (1, 5, 10, 15 mol.%). At first, the phase transition behavior of these neat microgels is characterized by DLS, DSC, and NMR.

DLS data yield the hydrodynamic radius *R*_h_. Figure S1 shows the temperature-dependent swelling curves for µ-PNiPAM/x-BIS, fitted by sigmoidal functions; see details in the Materials and Methods section. Typically, sigmoidal swelling curves are expected, proving the successful synthesis of microgel particles and their low polydispersity.

Particles with low crosslinker content (1 mol%) yield the largest structures in their swollen and the smallest structures in their de-swollen state. In contrast, particles with high crosslinker content (15 mol%) yield the smallest hydrodynamic radii in the swollen state, but the largest ones in the collapsed state. Thus, the low-*T* hydrodynamic radii decrease with increasing crosslinker content, while the high-*T* radii increase. Similar trends have been observed in previous DLS measurements [44,47]. These trends can be attributed to the collapse of less crosslinked outer structures onto a more rigid, highly crosslinked core, since the reaction rate of the BIS crosslinker is larger than that of NiPAM monomers [48,49]. An increase of *R*_h_ of the collapsed state with BIS content results from an increasing size of the highly crosslinked, rigid cores. On the other hand, less crosslinked structures offer larger swelling ratios $R_{\mathrm{sw}}=\frac{R_{h,\;\max}}{R_{h,\;\min}}$; see Figure S2. The observed swelling curve trends are in good agreement with literature [50,51].

Furthermore, the swelling curves reveal a monotonous shift of the VPTT to higher temperatures as the BIS content increases. The transition temperatures range from 31.8 °C (x = 1%) up to 34.4 °C (x = 15%), indicating a total shift of the inflection point by 2.6 °C; see Table S1 for a detailed summary of the DLS results. The general trend is consistent with literature [47]; see the comparison in SI. In addition, the transition widths are qualitatively evident from the slope of the fit function at the VPTT. A lower crosslinker content yields sharper transitions.

**Figure 1:**
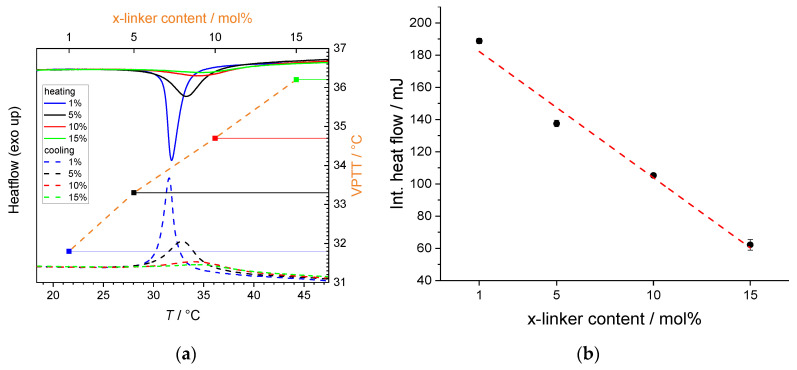
(**a**) DSC thermograms (heating: solid lines; cooling: dashed lines) of neat µ-PNiPAM microgels (1 wt.% in H_2_O) with different crosslinker content (left and bottom axis) and VPTT (squares, orange axis labels) as extracted from heating curves. Lines between squares are guides to the eye. A constant offset is subtracted from the heat flow to adjust for baseline differences. (**b**) Integrated heat flow for the phase transitions of neat microgels during heating cycle for different crosslinker contents.

DSC thermograms of the neat *µ*-PNiPAM/x-BIS microgels are shown in Figure 1a for the crosslinker content from 1 mol% up to 15 mol%. The phase transition from the swollen to the collapsed state is characterized by an endothermic heat flow. The less crosslinked microgel (1 mol%) shows the sharpest phase transition. As the crosslinking is increased, the endothermic peak is broadened significantly and the VPTT is increased. Both the shift in VPTT and the broadening are visible in the heating and cooling curves. As the cooling curves characterize the reswelling processes, exothermic heat flows are detected. The order of the VPTT is the same as in the heating curves. Hysteresis is not very pronounced at the cooling rates of 0.5 K/min employed here.

The integrated endothermic heat flow from the heating cycles is shown in Figure 1b in dependence on the crosslinker content, revealing a monotonous decrease. A decrease of the transition heat might be qualitatively explained by the reduction of the remaining NiPAM co-monomer content, as the BIS co-monomers do not undergo a transition. However, the integrated heat flow is reduced by a factor of three, and when comparing the 15 BIS with the 1 BIS sample, the NiPAM co-monomer content is reduced by only 14 %mol. Thus, the mere reduction of the NIPAM content does not explain the large degree of heat flow reduction. There is rather a disproportional decrease of the heat flow, implying a strong reduction of the transition heat released per NiPAM monomer.

This effect can be understood as a consequence of a reduced solvent release at elevated crosslinker content. The observation of continuous phase transitions for microgels has previously been explained by different transition temperatures for individual meshes, with the crosslinker being inhomogeneously distributed [52]. As crosslinking leads to a cooperativity of the VPT by mechanically coupling meshes of polymer networks, the local collapse of a polymer chain can induce collapses in neighboring chains. Friesen et al. described swelling curves of differently crosslinked PNiPAM microgels using a modified Flory-Rehner model, employing a Hill-like equation-modeled polymer–solvent interaction parameter *χ* [47]. A linear decrease of the Hill parameter *ν* with increasing BIS content was interpreted as a linearly decreasing number of water molecules per network chain, which are cooperatively leaving the chain at the VPT. Thus, our finding of a linear decrease of the integrated transition heat with BIS content confirms the above model description.

Liquid state ^1^H-NMR spectra serve to characterize the dynamic transition. Microgel ^1^H signals are only visible in liquid state NMR, when dipolar interactions of polymer protons are averaged by fast isotropic motions. This is the case in the swollen state, while for *T* >> VPTT dynamically restricted “solid” spins lead to significantly broadened and rapidly relaxing signals. This contrast enables us to characterize the phase transition.

**Figure 2:**
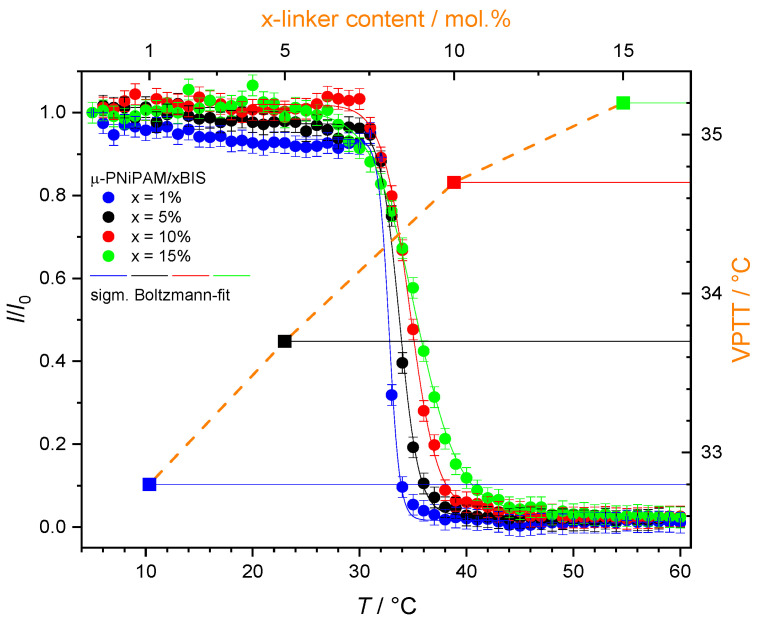
(i) Integrated normalized polymer signals in the range of *δ* = 1–3 ppm as acquired in temperature-dependent ^1^H-NMR spectra (circles; left and bottom axis, integrals are normalized on the lowest temperature) and (ii) extracted VPTTs (squares; right and top axis) for neat microgels containing different crosslinker content. Color code as indicated in the legend. Dashed line is guide to the eye. Solid lines are fits according to Equation (4).

Exemplary spectra of a neat microgel are shown in Figure S3. The reduction of the polymer resonances upon exceeding the VPTT is reflected by their integrals, which are given in Figure 2. The resulting sigmoidal curves are again fitted by Equation (4), yielding the VPTT as the inflection point; see Figure 2 and Table 1. At the inflection point, negative slopes decreasing with increasing crosslinker content are observed, confirming the increasing width of the transition seen in DSC and DLS data.

NMR liquid state intensities reveal a monotonous shift of the VPTT to higher values as a function of crosslinker content. The total VPTT shift amounts to 2.4 °C between the lowest and the highest crosslinker density.

### 2.2. Discussion of Influence of Crosslinker Content on Volume Phase Transition

**Figure 3:**
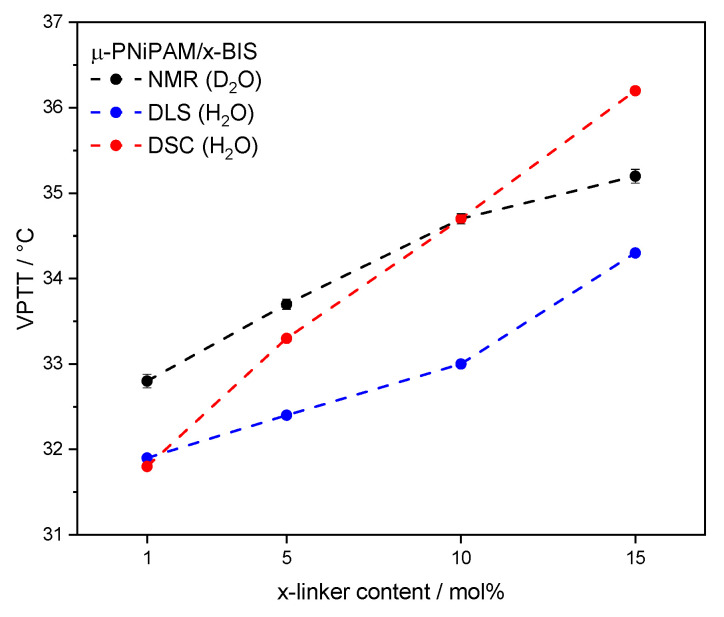
Plot of VPTT determined by different methods for neat microgels containing different amounts of crosslinker.

Figure 3 summarizes the VPTT values for the differently crosslinked neat microgels extracted from each method. With increasing crosslinker content a monotonous shift of the VPTT towards higher values is observed. As this trend is clearly visible, all methods are in good agreement and consistent.

Note that samples for DSC and DLS are prepared in H_2_O, while samples for NMR rely on the use of D_2_O as a solvent. It is known that the use of D_2_O increases the VPTT by about 1–2 °C [53], since the deuterium bond to the amide groups is stronger than corresponding hydrogen bonds [53,54]. In view of this expected shift, the agreement in Figure 3 at 1% crosslinker content is excellent.

Differences arise at higher crosslinker content due to the fact that in different methods the VPTT reflects different physical properties. In DLS the smallest VPTT difference between the 1BIS and 15BIS microgel is observed, in contrast to the results from DSC, which show the largest temperature range of all applied methods.

Generally, the phase transition shift towards higher temperatures can be explained with the chemical nature of the crosslinker itself. As the latter is more hydrophilic than the co-monomer NiPAM, an increasing content of BIS will raise the overall hydrophilicity of the microgel by introducing a significant amount of additional amide groups. Hence, more hydrogen bonding between polymer and solvent is possible, stabilizing the swollen state. An additional contribution to the VPTT enhancement may arise from the decreasing mesh size as the crosslinker content is increased. As the average degree of polymerization between crosslinks is decreased, according to Wu et al., the VPTT is shifted upwards [52].

Concerning the controversy in literature about the influence of crosslinker content on the VPTT, we could here confirm the increase seen by Friesen et al. [47] and could verify their model by the finding of linearly decreasing transition heats. Furthermore, the results of all methods show that the structural, the dynamic, and the calorimetric transition are consistently shifted upwards with increasing crosslinker content, but to a slightly different degree.

### 2.3. Additive Influence on Phase Transition

In order to investigate the effects of the aromatic additives on the polymer phase transition, the shift Δ*T* of the VPTT, which is caused by the additive, is defined as

(1) $$\Delta T={\mathrm{VPTT}}_{Polymer+Additive}-{\mathrm{VPTT}}_{Polymer}$$

Temperature-dependent swelling curves from DLS and their fits, DSC thermograms, and temperature-dependent ^1^H NMR integrals with their fits and the resulting VPTT depression for each method are given in the SI, Figures S4–S14. Figure 4 summarizes the VPTT temperature shifts Δ*T* for solutions containing *m*-HBA (Figure 4a) and 2,4-DHBA (Figure 4b) as a function of crosslinker content.

Microgel dispersions containing either additive show a VPTT, which is generally shifted to lower temperatures in each of the methods, as indicated by negative Δ*T* values. While the absolute values of Δ*T* differ significantly for both additives, several similarities of the trends occur, which suggest generic behavior.

**Figure 4:**
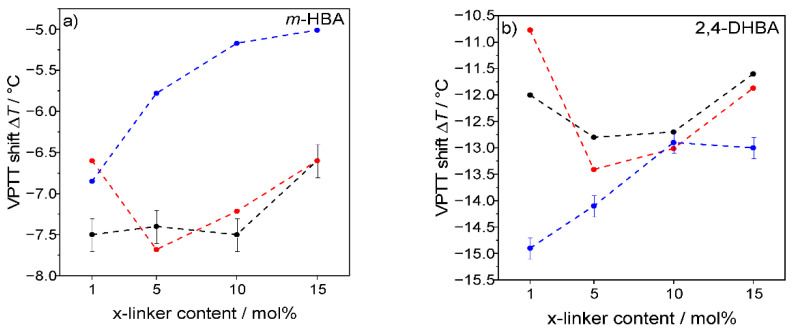
VPTT shift Δ*T* for different methods as a function of crosslinker content, as induced by the presence of (**a**) 20 mM *m*-HBA and (**b**) 2,4-DHBA as additives. Microgel concentrations are 1 wt.% (1 × 10^−^^3^ wt.% for DLS). Error bars reflect the standard deviation. The color code corresponds to different methods (DSC = red; DLS = blue; NMR = black).

A comparison of the Δ*T* patterns between both additives, considering each method separately, reveals that they are similar to each other, only shifted along the Δ*T* axis. ^1^H-NMR Δ*T* shifts are in the range of those from DSC; see red and black data points in Figure 4. A general feature is a local minimum in case of data from DSC and NMR, and a monotonous increase of Δ*T* in case of DLS. However, the local minimum in phase transition temperature for the 5 mol% gel with 2,4-DHBA is more pronounced compared to the polymer-additive system with *m*-HBA, where errors in particular for the NMR data are too large to yield a clear trend, while a higher precision due to generally larger shifts is achieved in case of 2,4-DHBA. We thus conclude on a higher responsivity of the microgel containing 5 mol% of BIS crosslinker towards additives as compared to microgels with lower or higher crosslinking density.

DLS measurements, reflecting the hydrodynamic radius *R*_h_ as a macroscopic size parameter, reveal a distinct monotonous decrease in |Δ*T|* with increasing crosslinker content. Thus, in loosely crosslinked microgels, the effect of an additive on the microgel particle size is most pronounced. Possibly, outer segments of the loosely crosslinked microgels are affected by pre-transition aggregation, induced by favorable interaction with the additive, causing a reduction of the hydrodynamic radius already at lower temperatures. This might cause the structural transition to occur at slightly lower temperatures than the calorimetric and the dynamic transitions. It also explains a larger influence of the additive on the VPTT at low BIS content and thus the monotonous decrease of |Δ*T|*.

Higher responsivity at intermediate crosslinker content, as seen for the calorimetric and dynamic transitions, may be an effect of mesh sizes in the microgel matrix being influenced by the NiPAM:BIS ratio. In general, mesh sizes decrease as more crosslinker is used. As mesh sizes approach the hydrodynamic radii of applied additives, the polymer matrix may, on the one hand, hinder the diffusion of such additive molecules [55]. On the other hand, specific distances between functional groups in the polymer may be beneficial for the steric demand of the additive, which might foster the ability to effectively form polymer-additive hydrogen bonds. We assume that the efficiency to form the latter is coupled with large values of |Δ*T|* as a large number of additive molecules are arranged spatially close to the polymer, inducing the hydrophobic collapse.

### 2.4. NMR—Incorporated Species and Dynamics

**Figure 5:**
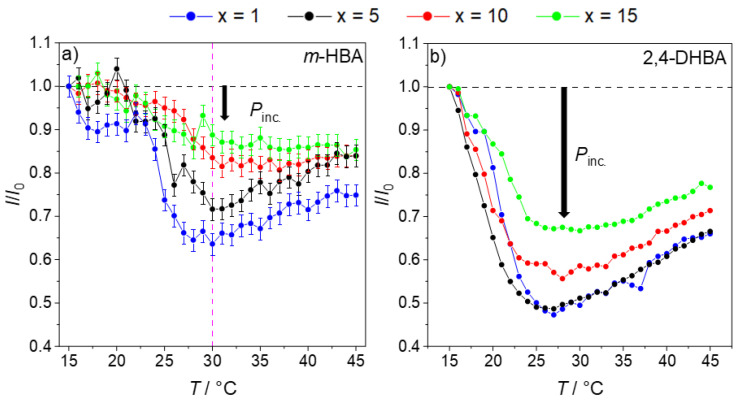
Integrated aldehyde proton signals from temperature dependent ^1^H spectra (15 to 45 °C in 1 °C steps) for 20 mM of (**a**) *m*-HBA and (**b**) 2,4-DHBA in the presence of microgels using identical acquisition parameters. Integrals are normalized to the lowest temperature. The arrow indicates the maximum of *P_inc_* for 15% BIS.

In addition to the polymer ^1^H NMR signals (given in Figure 2), the additive resonances can be evaluated in a similar manner, as suggested in previous work [56]. Figure 5 shows the integrated aldehyde proton signals of the additive in dependence on temperature for different crosslinker content. At low temperatures *T* < VPTT, the aldehyde signal originates from freely diffusing additive species and loosely bound species, interacting with the polymer. As the temperature is increased and induces the microgel to collapse, a partial signal loss is detectable at *T* ≈ VPTT. At temperatures *T* >> VPTT, the additives signal recovers to some extent.

The relative additive signal loss corresponds to a fraction of incorporated additive *P_inc_*, which is not detectable under liquid state conditions [56]. It can be calculated by comparing the integrals of a temperature series with identical acquisition settings as follows:

(2) $$P_{inc}=1-\frac{I_{\left(T\right)}}{I_{0}}$$

Residual proton signals arise from a fraction *P_free_* = 1 − *P_inc_* of mobile additives in the solution state. These may include free additives in solution as well as additives interacting with the outer microgel interface but not being immobilized. We note that the process of additive incorporation is fully reversible, since after cooling the dispersion, inducing microgel re-swelling, previously incorporated additive species are released (see Figure S15).

The decrease of the additive integral around the VPTT occurs in all combinations of microgels with additives and suggests that all microgel structures incorporate a fraction of the additive during the phase transition process. Remarkably, there is a clear trend with crosslinker density, as *P_inc_* is higher for less crosslinked microgels, which may be caused by a larger swelling factor *R*_sw_ (see Figure S2) and therefore higher flexibility to incorporate additive molecules. For example, in the case of *m*-HBA in 1%-microgels at 30 °C (see dashed purple line in Figure 5a intersecting the blue curve), a minimum in signal intensity occurs. Here, a maximum fraction *P_inc_* = 36% of the additive is immobilized inside collapsed microgel structures. This value agrees very well to that for *m*-HBA incorporation into collapsed homopolymer particles of PNIPAM, i.e., 33% [56].

Surprisingly, while in PNIPAM homopolymer solutions a monotonous decrease of the additive intensity finally reaches a plateau value at high temperature [56], the microgels mostly show a maximum of incorporation. When the temperature is raised above 30 °C, *P_inc_* decreases for the 1- and 5-BIS microgels, which leads to the conclusion that the initially trapped additive molecules are partially released again upon further heating. Comparing aldehyde integrals to polymer integrals reveals that the phase transition is not fully completed at *T* = 30 °C, which leads to a “sponge-like” effect. During the first phase of collapse (up to about 30°C), the additive, which is interacting with the polymer, is immobilized, but upon further collapse of the microgel, it is partially squeezed out from the microgel particle. This release in the second phase of collapse is less pronounced for highly crosslinked microgels. We tested whether the squeeze out is a time-dependent effect but found that the incorporated fraction is constant after a temperature jump; see Figure S16. Thus, the temperature-dependent incorporated fractions represent a thermodynamic equilibrium.

Figure 5b shows similar trends for 2,4-DHBA; however, incorporated fractions tend to be larger, namely up to 53% for the 1- and 5-BIS microgel. The “sponge-like” effect is even more clearly visible in the data for 2,4-DHBA.

**Figure 6:**
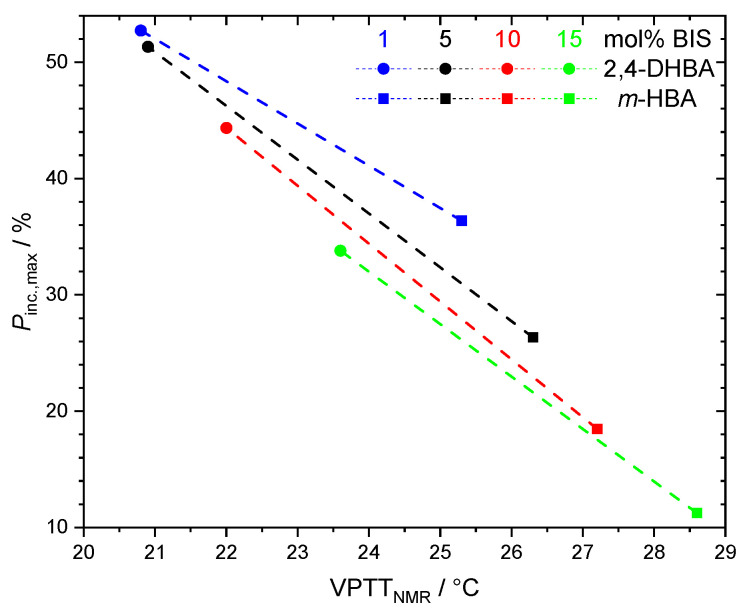
Maximum of incorporated species *P_inc,max_* as a function of the transition temperature VPTT_NMR_ for 2,4-DHBA (circles) and *m*-HBA (squares) for different crosslinker contents (blue x = 1, black x = 5, red x = 10, green x = 15). Dashed lines are guide to the eye and connect polymers of the same crosslinker content.

Considering data from both additives, the amount of incorporated species *P_inc,max_* correlates qualitatively with the VPTT (see Figure 6). A large VPTT reduction corresponds to a large amount of incorporated species. It can be assumed that a large VPTT reduction is caused by breaking up hydrogen bonds between the polymer and solvent. Hence, a large number of additive molecules must be present in local vicinity to the polymer. As *P_inc,max_* is larger for 2,4-DHBA, it can be assumed that the overall additive–polymer interaction is stronger in comparison to *m*-HBA, a fact that agrees well with the larger VPTT shift induced by 2,4-DHBA; see Figure 6. It is interesting to note that the correlation of *P_inc,max_* with VPTT is observed here upon variation of the crosslinker content, while a similar correlation was previously found upon variation of a larger selection of additives [56].

### 2.5. Additive Dynamics

To investigate local additive dynamics and distribution, the transversal spin relaxation time *T*_2_ is measured in a temperature series for all microgels containing *m*-HBA. ^1^H relaxation times *T*_2_ of the aldehyde proton signal are converted into corresponding relaxation rates *R*_2_ according to

(3) $$R_{2}=\frac{1}{T_{2}}$$

**Figure 7:**
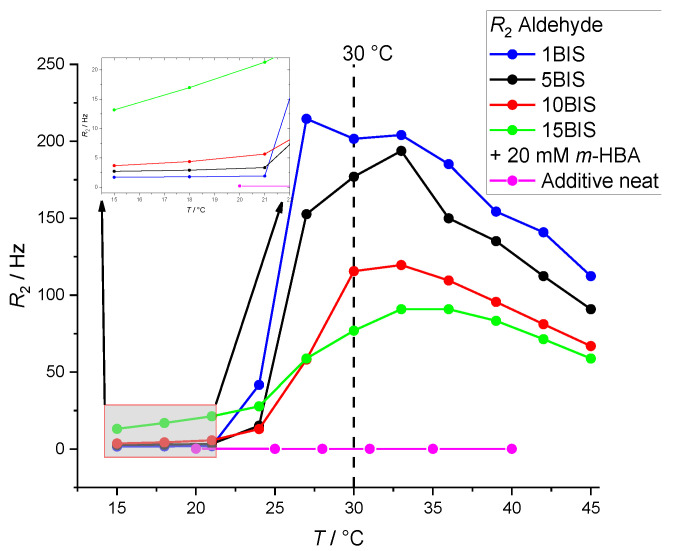
Transversal relaxation rates of aldehyde protons from 20 mM *m-*HBA additive without (pink) and with microgel in a temperature series. Lines are guides to the eye.

They are displayed in Figure 7. Generally, exponential decays are observed, yielding one distinct relaxation rate. As a reference, relaxation rates for a 20 mM solution of *m*-HBA in D_2_O in absence of any polymer are given.

The relaxation rate *R*_2_ can be understood as a qualitative measure of molecular immobilization. As Figure 7 indicates, the additive reference solution shows small relaxation rates of 0.14–0.20 Hz throughout the whole temperature range. Relaxation rates for *m*-HBA in presence of microgels are enhanced in the low temperature regime at *T* < VPTT, revealing an interaction between aldehyde protons and microgels (see Figure 7, magnification). Here, *R*_2_ of the aldehyde protons is increasing from 2 Hz (1BIS) up to 13 Hz (15BIS), indicating an additive immobilization, which increases with increasing crosslinker content.

As the temperature is raised to the VPTT region, *R*_2_ shows a maximum followed by a decrease. Considering the maximum *R*_2_ values, the trend in dependence on crosslinker density is inverted. The collapsed 1BIS microgel undergoes strongest interactions (*R*_2, 30°C_ = 202 Hz) with the additive, while in the 15BIS samples relaxation rates are much smaller (*R*_2, 30°C_ = 77 Hz).

In the higher temperature regime, *T* > VPTT, the relaxation rates decrease again. This behavior correlates with the increasing aldehyde proton integrals (Figure 5a) and supports the interpretation of a “sponge-like” effect, as in the second phase of the collapse additive molecules are squeezed out of the particle interior, thus gaining mobility and reducing *R_2_*. We note here that the exponentiality of all relaxation data implies a fast exchange averaging between all additive localizations, which are detectable. In conclusion, the detectable fraction *P_free_* consists of (a) free species and (b) detectable incorporated species, with the latter contributing with a larger relaxation rate to the measured average value of *R_2_*.

The sponge-like effect found in microgels is an interesting feature, which clearly differs from the additive interactions with PNiPAM homopolymers, where temperature-dependent incorporated fractions generally show a simple monotonous behavior of the incorporated fractions with a plateau above the critical temperature [56]. In microgels, the sponge effect consists of an initial immobilization of a large number of additive molecules, part of which are re-mobilized at higher temperatures by being squeezed out of the collapsing microgel as temperature is further enhanced. A low crosslinking density favors the sponge effect, probably mainly because a less dense network facilitates the uptake of the additive in the initial stage of the phase transition.

## 3. Conclusions

A systematic dependency of the VPTT on the crosslinker content in µ-PNiPAM/x-BIS microgels is found. VPTT values extracted from DLS, DSC, and ^1^H NMR confirm a consistent shift to higher temperatures with increasing crosslinker content, while the VPTT values differ slightly for each technique. This shift of the VPTT has to be considered in drug delivery applications, while it does not imply an optimum value of the crosslinker density.

Small aromatic additives decrease the neat microgel VPTT, again confirmed by DLS, DSC, and ^1^H NMR. The ability to induce the collapse of a microgel is dependent on the isomer structure, as Δ*T* is markedly different for each additive. For both additives a joint pattern emerges, which shows that the calorimetric transition observed in DSC is most affected in x = 5% microgels, while the structural transition, as manifested by changes of the hydrodynamic radius, is most sensitive towards an additive at the lowest crosslinker content of 1%. For different systems, varying x, or additive structures, the maximum incorporated fraction *P*_inc,max_ correlates with Δ*T*. In conclusion, an optimum crosslinker density for drug delivery has to be a compromise balancing the influence of the drug and that of the crosslinker density: while microgels with low crosslinker density result in a larger drug incorporation, they simultaneously suffer from the largest VPTT shift. In addition, the distinct drug chemical structure will have a significant influence on both effects, which have to be characterized for each specific drug structure.

^1^H-NMR spectra of *m*-DHB yield a quantification of additive incorporation. Additive molecules incorporated during the initial stages of microgel collapse (*T* ≈ VPTT) are partially released upon advancing collapse (*T* > VPTT). This squeeze out effect bears potential for application, since microgels may be loaded with a large amount of drug at a temperature around VPTT, while the drug will be released upon raising the temperature. This feature is unique to microgels and is not observed in PNiPAM homopolymer samples.

## 4. Materials and Methods

### 4.1. Chemicals

**Scheme 1:**
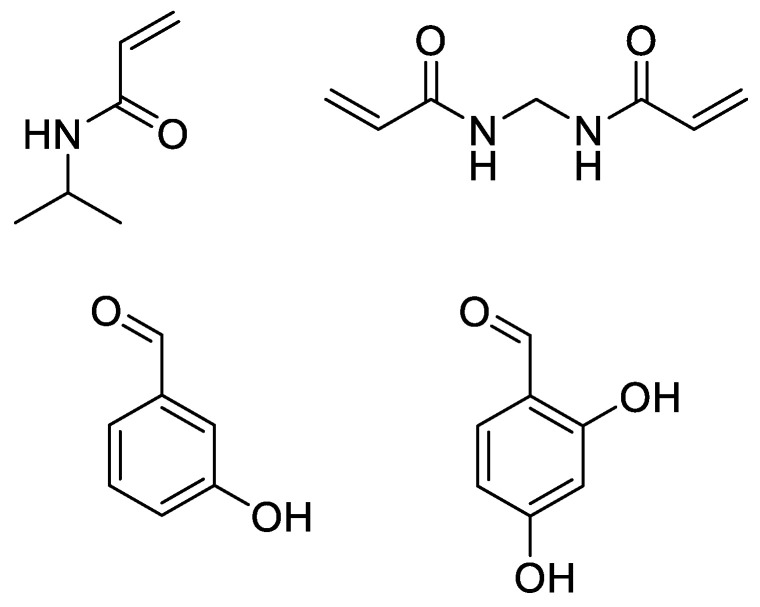
Monomers N-isopropylacrylamide (NiPAM) (upper left) and bifunctional crosslinker *N*,*N*′-Methylenebis(acrylamide) (BIS; upper right) used for microgel synthesis. Aromatic model drugs 3-Hydroxybenzaldehyde (*m*-HBA; lower left) and 2,4-Dihydroxybenzaldehyde (2,4-DHBA; lower right) used as additives.

Structures of the monomers for microgel synthesis are given in Scheme 1. *N*-isopropylacrylamide (NiPAM, ≥99%) and *N*,*N*′-Methylenebis(acrylamide) (BIS, >99%) were purchased from Sigma Aldrich and used without further purification. Sodium dodecyl sulfate (SDS, >99%) was purchased from Fluka. All samples were prepared in ultrapure water (MilliQ, Merck, Darmstadt, Germany) with a resistance of >18 MΩ. For NMR solutions, D_2_O (99.9 atom %, Sigma Aldrich, St. Louis, MO, USA) was used instead. Solutions were prepared immediately before use to prevent possible oxidation reactions at the additive’s functional groups. Additive incorporation into microgels was performed by mixing additive stock solutions and microgel dispersions at room temperature to achieve a final additive concentration of 20 mM. This additive concentration, chosen as a compromise of sufficient effects on the VPTT and limited additive solubilities, is identical for all experimental methods.

### 4.2. Synthesis

Poly(*N*-isopropylacrylamide) microgels (µ-PNiPAM) were synthesized by a conventional precipitation polymerization with sodium dodecyl sulfate (SDS) as surfactant [57], employing four different crosslinker amounts. Synthesis was performed in 500 mL three-neck flasks equipped with a reflux condenser, Pt-100 thermometer, and a nitrogen inlet. The flask was thermally isolated and heated using an oil bath on a magnetic stirrer. Previously ultrasonicated and degassed ultrapure water was used as solvent. Then, 380 mL of solvent was heated to 70 °C under continuous stirring while using a glass inlet for degassing with nitrogen in order to remove residual oxygen. The solvent temperature was equilibrated for at least 30 min, then SDS (75.4 mg; 2.6 × 10^−4^ mol) was added. After complete dissolution of SDS, the monomer NiPAM (49.48 mM) and the crosslinker BIS (1, 5, 10, and 15 mol.%, with respect to the initial molar amount of NiPAM) were added to the SDS solution with an additional 18 mL of solvent. The solution was kept at 70 °C for 1 h to ensure dissolution and thermal equilibration. Initiation was done by injecting a solution of potassium persulfate (KPS, 100 mg in 2 mL water) into the flask. The reaction was left to proceed for 4 h at 70 °C under constant stirring (650 rpm). All solutions turned turbid after the first 2–10 min after KPS injection, indicating the process of radical polymerization and particle formation. After reaction, the solution was cooled down to room temperature and stirred overnight. Purification was done by at least five centrifugation/redispersion processes. The microgels were then lyophilized. Stock solutions were prepared from dried samples.

### 4.3. Differential Scanning Calorimetry (DSC)

DSC data were acquired on a *Micro DSC III* Calorimeter (Setaram, Caluire, France) in four consecutive heating/cooling cycles from 5 °C to 60 °C and back to 5 °C, employing a heating rate of 0.5 °C/min and water in the reference cell. The last three heating ramps were analyzed. Concentrations were 1 wt.% of polymer material and 20 mM of additive. The VPTT is defined as the temperature where the largest exothermic heat flow in a heating curve is observed, which is usually a global minimum in the thermogram. We defined the transition width as twice the difference of VPTT and onset temperature.

### 4.4. Dynamic Light Scattering (DLS)

DLS measurements were performed using a standard goniometer setup (ALV/CGS-3, ALV GmbH, Langen, Germany) with toluene in the matching bath. The light source was a HeNe laser (λ = 632.8 nm, 35 mW output power). The temperature was controlled based on the goniometers built-in sensor and an external thermostat, yielding a stability of ±0.05 °C.

Samples for DLS were prepared with a polymer concentration of 1 × 10^−3^ wt.% in water. Stock solutions of additives were filtrated using a 0.45 µm cellulose acetate syringe filter. Prior to each run, the microgels were stored at 8 °C for at least 24 h, then temperature-dependent swelling curves were recorded beginning at the lowest temperature. For each temperature step, equilibration was allowed for at least 20 min to reach an equilibrium state. For each temperature, at least five autocorrelation functions were recorded at a scattering angle of *θ* = 90° and a correlation time of at least 60 s.

The VPTT in DLS is defined as the inflection point of an empirical fit function, namely a sigmoidal Boltzmann function (OriginLab, OriginPro 2021), which describes the hydrodynamic radius *R*_h_ in the transition region:

(4) $$R_{h}{}_{\left(T\right)}=\frac{A_{1}-A_{2}}{1+e^{\left(T-T_{0}\right)/dT}}+A_{2}$$

The upper (low *T*) and lower (high *T*) plateaus are described by *A*_1_ and *A*_2_, respectively. The inflection point is given as $T_{0}$.

### 4.5. Nuclear Magnetic Resonance Spectroscopy (NMR)

Temperature-dependent ^1^H spectra were taken on a Avance NEO 400 MHz NMR spectrometer (Bruker, Rheinstetten, Germany) equipped with a BB probe head (Bruker, Rheinstetten, Germany). The temperature was controlled using an air stream with the temperature adjusted by an external cooling system (BCU II, Bruker, Rheinstetten, Germany).

Temperature-dependent transverse relaxation time (*T*_2_) measurements were conducted on a Bruker Avance III HD 400 MHz NMR spectrometer equipped with a gradient probe head with a selective ^1^H insert (“Diff50”, Bruker, Rheinstetten, Germany). The temperature was controlled in the same way as described above, in this case using an in-house design air cooling device. *T*_2_ relaxation measurements were accomplished by the Carr-Purcell-Meiboom-Gill (CPMG) pulse sequence with a fixed echo time of *τ* = 30 ms.

In each experiment series data were taken from low to high temperatures. Directly beforehand, the built-in temperature sensor was calibrated by determining the sample temperature in a reference tube containing a Pt100 thermocouple in oil. The 1D 1H spectra series and T2 relaxation measurements were acquired using 15 min of equilibration time following each temperature change. The shim and other parameters were kept constant; however, the automatic tuning and matching accessory (ATMA) was re-employed for each temperature step.

## Figures and Tables

**Table 1:** Fit results of ^1^H NMR intensities for neat µ-PNiPAM/x-BIS microgels in D_2_O.

	VPTT_NMR_/°C	Slope at VPTT /°C^−1^
µ-PNiPAM/1BIS	32.8	−1.39
µ-PNiPAM/5BIS	33.7	−0.64
µ-PNiPAM/10BIS	34.7	−0.33
µ-PNiPAM/15BIS	35.2	−0.18

## Data Availability

Data can be obtained from the authors upon request.

## Supplementary Materials

The following supporting information can be downloaded at: https://www.mdpi.com/article/10.3390/gels8090571/s1. Figure S1. Temperature-dependent hydrodynamic radii R_h_ of microgel structures containing different amounts of crosslinker. Figure S2. Swelling ratios R_sw_ for differently crosslinked microgels. Figure S3. ^1^H-NMR temperature series of a microgel acquired with equal spectral parameters. Table S1. Summary of DLS data for neat microgels in H_2_O and comparison with reported data from literature. Figure S4. Plot of the VPTT (polymer only; with additive) and calculated ΔT from DSC against the crosslinker content for *m*-HBA. Figure S5. Plot of the VPTT (polymer only; with additive) and calculated Δ*T* from DSC against the crosslinker content for 2,4-DHBA. Figure S6. Calculated microgel VPTT depression Δ*T* from DSC data in presence of two different aromatic additive structures. Figure S7. Temperature-dependent hydrodynamic radii R_h_ of microgel particles (neat microgel and with *m*-HBA) containing different amounts of crosslinker. Figure S8. Temperature-dependent hydrodynamic radii *R*_h_ of microgel particles (neat microgel and with 2,4-DHBA) containing different amounts of crosslinker. Figure S9. VPTT (neat microgel and with *m-*HBA) and calculated Δ*T* from DLS against crosslinker content. Figure S10. Plot of the VPTT (neat microgel and with 2,4-DHBA) and calculated Δ*T* from DLS against crosslinker content. Figure S11. Microgel VPTT depression Δ*T* from DLS data in presence of two different aromatic additive structures. Figure S12. Integrated normalized polymer signals as acquired in ^1^H-NMR temperature series for neat microgels containing different crosslinker content and 20 mM of *m*-HBA. Figure S13. VPTT (neat microgel and with *m*-HBA) and calculated Δ*T* from NMR against the crosslinker content. Figure S14. VPTT (neat microgel and with 2,4-DHBA) and calculated Δ*T* (red squares) from NMR against crosslinker content. Figure S15. Integrated aldehyde signal for µ-PNiPAM/1-BIS with 20 mM *m*-HBA for a series of temperature jumps. Figure S16. Integrated aldehyde signal in dependency of time and temperature for µ-PNiPAM/1-BIS with 20 mM *m*-HBA.
