# Supplementary material for: Interplay of the Influence of Crosslinker Content and Model Drugs on the Phase Transition of Thermoresponsive PNiPAM-BIS Microgels
Source: Gels. 2022 Sep 8;8(9):571. doi: 10.3390/gels8090571 (PMC9498534; doi:10.3390/gels8090571)
Supplement: Supplementary file 1 [file gels-08-00571-s001.zip › gels-1883790-supplementary.pdf]

gels

Article

Interplay of the Influence of Crosslinker Content and Model Drugs on the Phase Transition of Thermoresponsive PNiPAM-BIS Microgels

Daniel Schlattmann and Monika Schönhoff *

Institute of Physical Chemistry, University of Münster, Corrensstr. 28-30, 48149 Münster, Germany
* Correspondence: schonhoff@uni-muenster.de

Citation: Schlattmann, D.; Schönhoff, M. Interplay of the Influence of Crosslinker Content and Model Drugs on the Phase Transition of Thermoresponsive PNiPAM-BIS Microgels. *Gels* **2022**, *8*, 571. https://doi.org/10.3390/gels8090571

Academic Editors: Hailei Zhang and Yonggang Wu

Received: 9 August 2022
Accepted: 5 September 2022
Published: 8 September 2022

Publisher's Note: MDPI stays neutral with regard to jurisdictional claims in published maps and institutional affiliations.

Copyright: © 2022 by the authors. Licensee MDPI, Basel, Switzerland. This article is an open access article distributed under the terms and conditions of the Creative Commons Attribution (CC BY) license (https://creativecommons.org/licenses/by/4.0/).

Supplementary Materials

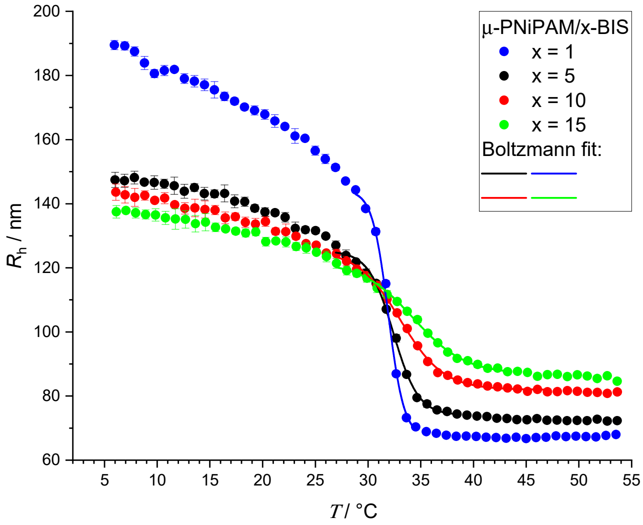

Figure S1. Temperature dependent hydrodynamic radii R_h of microgel structures containing different amounts of crosslinker. Results shown from a heating cycle starting at low temperatures. Lines are fits according to equation (4).

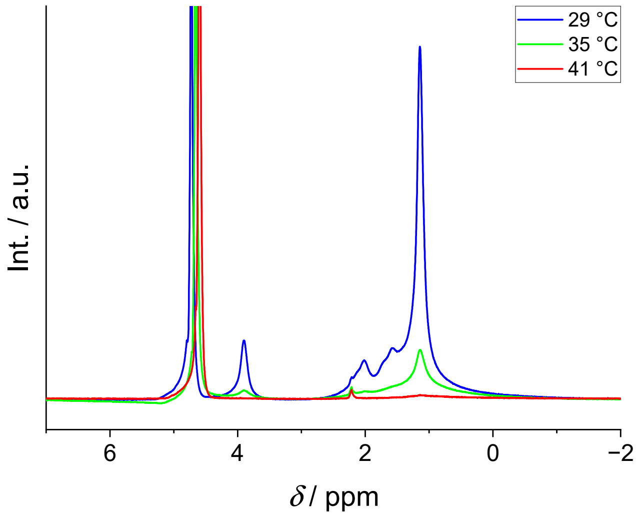

Figure S2. ^{1}H-NMR temperature series of a microgel acquired with equal spectral parameters.

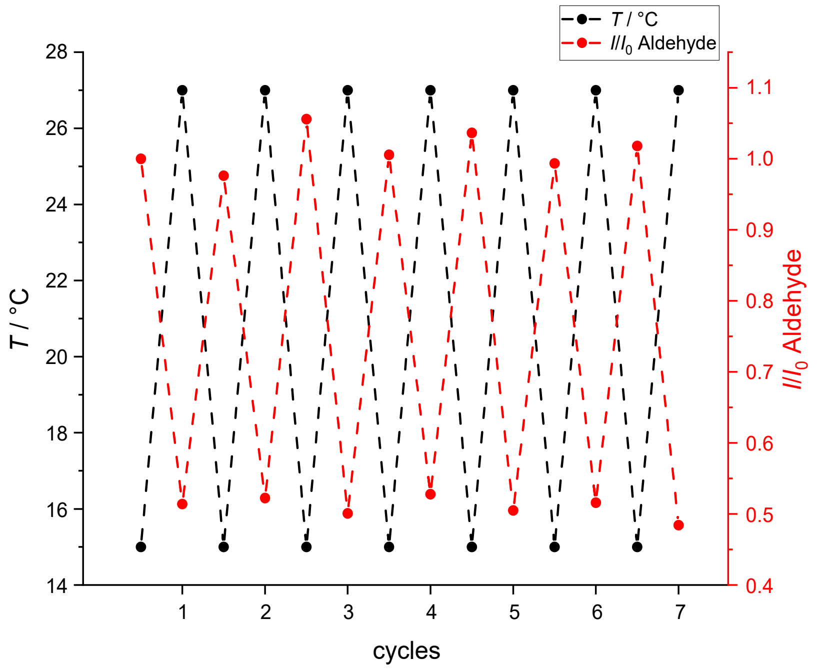

Figure S3. Integrated aldehyde ^{1}H signal for µ-PNiPAM/1-BIS with 20 mM *m*-HBA for a series of temperature jumps. Integrals are normalized to the value measured at 15 °C.

It could be shown, that the incorporation of additive is fully reversible and can be accomplished in the investigated time intervals.

NMR also revealed that incorporation of additives is *not* time dependent in the investigated period of time (2.5 h). Integrals are normalized to 15 °C. Equilibration time was 30 min at least. Data show no significant deviation from reached value at 27 °C and is forming a plateau, indicating no further reduction of $P_{inc.}$ with time:

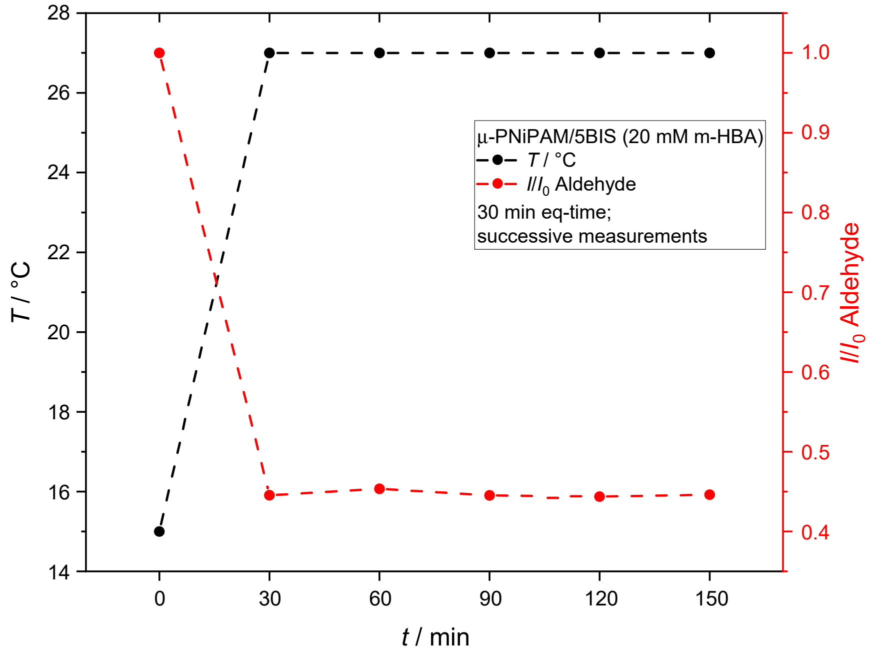

Figure S4. Integrated aldehyde signal for µ-PNiPAM/1-BIS with 20 mM *m*-HBA in dependency of time, following a temperature jump.

DSC

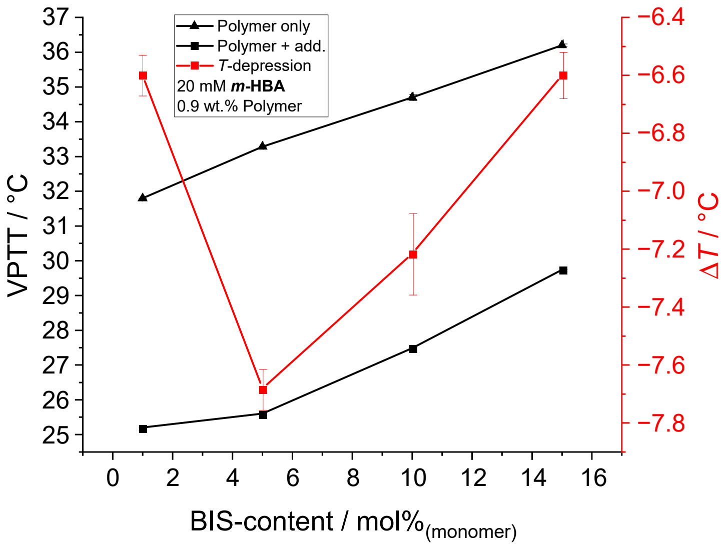

Figure S5. Plot of the VPTT (triangles: polymer only, black squares: depressed temperature) and calculated ΔT (red squares) against the crosslinker content for *m*-HBA.

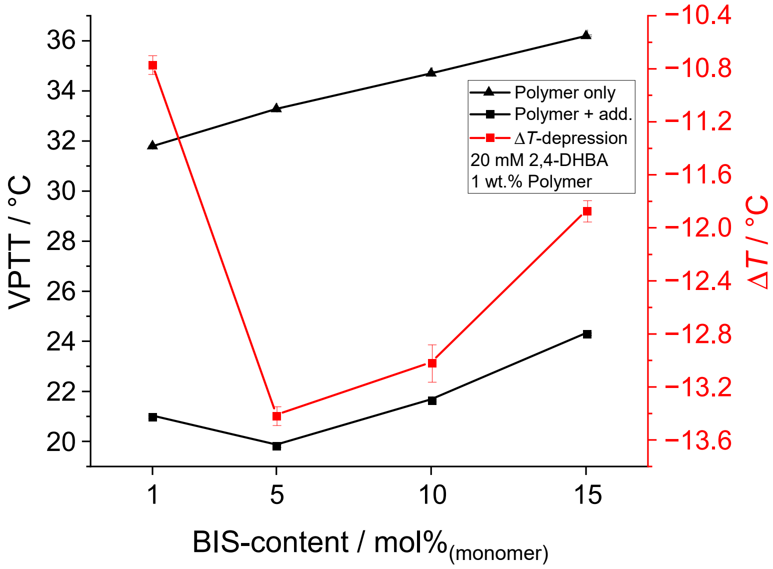

Figure S6. Plot of the VPTT (triangles: polymer only, black squares: depressed Temperature) and calculated ΔT (red squares) against the crosslinker content for 2,4-DHBA.

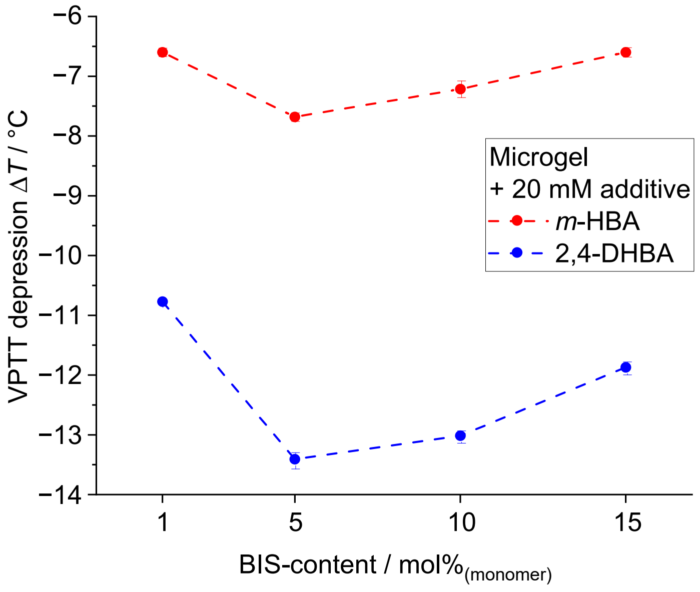

Figure S7. Calculated microgel VPTT depression Δ*T* from DSC data in presence of two different aromatic additive structures. Concentrations were 1 wt.% for the microgels and 20 mM for each additive. Error bars do reflect standard deviation. Colorcode corresponds to the microgels VPTT influenced by additives relative to each other (low *T* = blue; high *T* = red).

DLS

Table S1. Summary of DLS data for neat μ-PNiPAM/x-BIS microgels in H_2O and comparison with reported data from Friesen et al.

	$R_{h,15\ °C}$ / nm	$R_{h,50\ °C}$ / nm	$R_{sw,15°C/50°C}$	VPTT/°C	VPTT/°C	$PDI_{25°C}$ ± 0.02
μ-PNiPAM/1BIS	176	67	2.6	31.9	-	0.06
μ-PNiPAM/2.5BIS	-	-	-	-	34.1*	-
μ-PNiPAM/5BIS	143	72	2.0	32.4	35.7*	0.02
μ-PNiPAM/10BIS	138	81	1.7	33.0	37.1*	0.04
μ-PNiPAM/15BIS	132	87	1.5	34.3	38.6*	0.03

*Friesen, S., et al., Accounting for Cooperativity in the Thermotropic Volume Phase Transition of Smart Microgels. Gels, 2021. **7**(2): p. 42.

We note that absolute phase transition temperatures may differ, caused by different synthesis protocols. Nonetheless, temperature shifting trend remains the same.

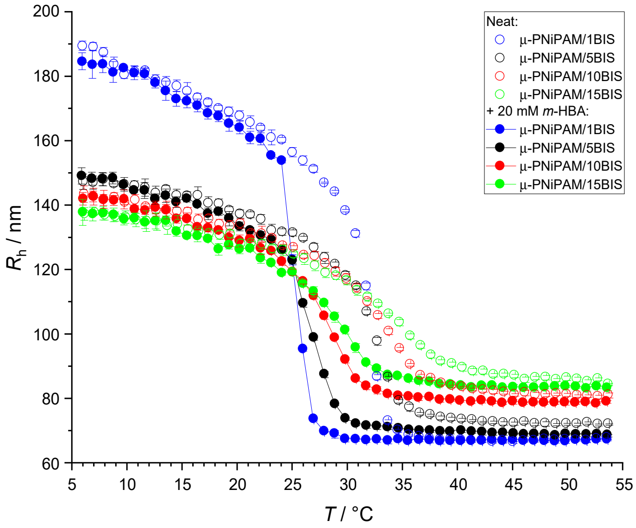

Figure S8. Temperature dependent hydrodynamic radii R_h of microgel particles containing different amounts of crosslinker (hollow circles: neat Polymer; solid circles: 20 mM *m*-HBA). Results shown from a heating cycle starting at low temperatures. Lines are guide to the eye.

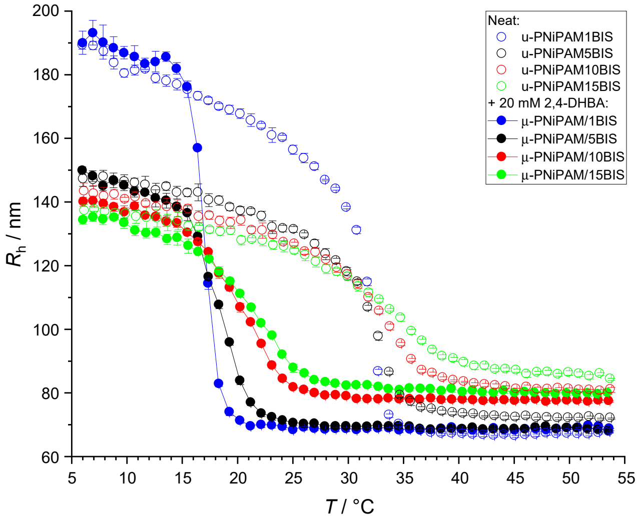

Figure S9. Temperature dependent hydrodynamic radii R_h of microgel particles containing different amounts of crosslinker (hollow circles: neat Polymer; solid circles: 20 mM 2,4-DHBA). Results shown from a heating cycle starting at low temperatures. Lines are guide to the eye.

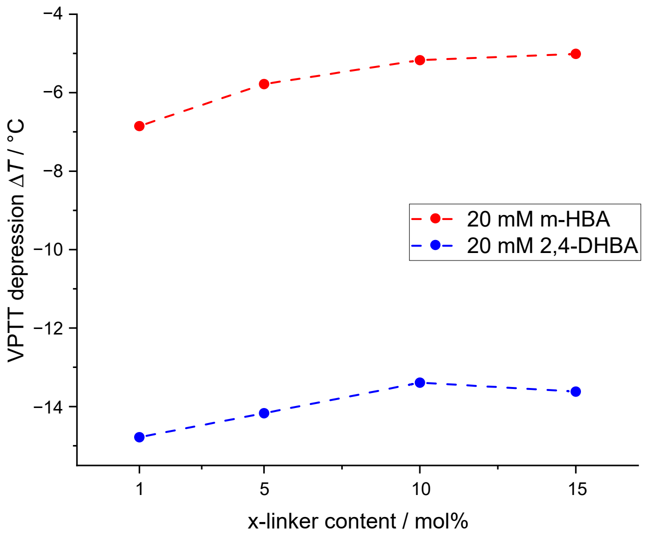

Figure S10. Calculated microgel VPTT depression Δ*T* from DLS data in presence of two different aromatic additive structures. Concentrations were 1 wt.% for the microgels and 20 mM for each additive. Error bars do reflect standard deviation. Colorcode corresponds to the microgels VPTT influenced by additives relative to each other (low *T* = blue; high *T* = red).

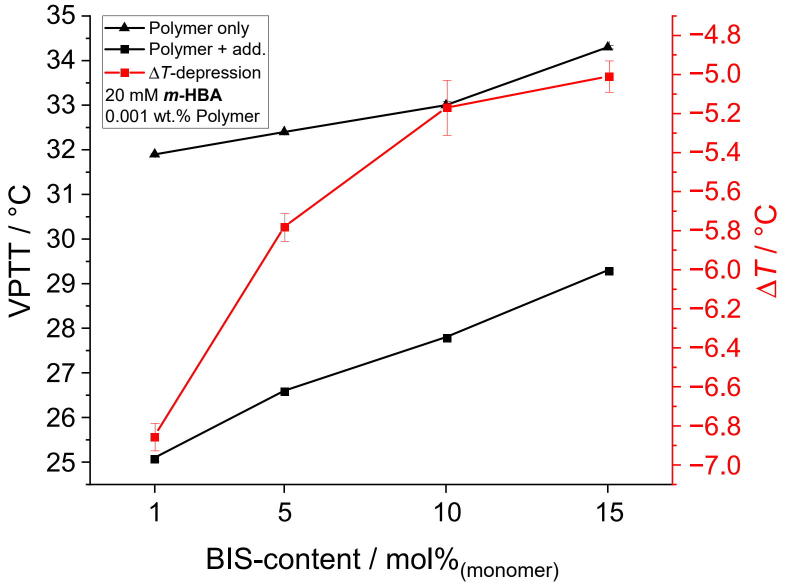

Figure S11. Plot of the VPTT (triangles: polymer only, black squares: depressed Temperature) and calculated Δ*T* (red squares) against the crosslinker content for *m*-HBA.

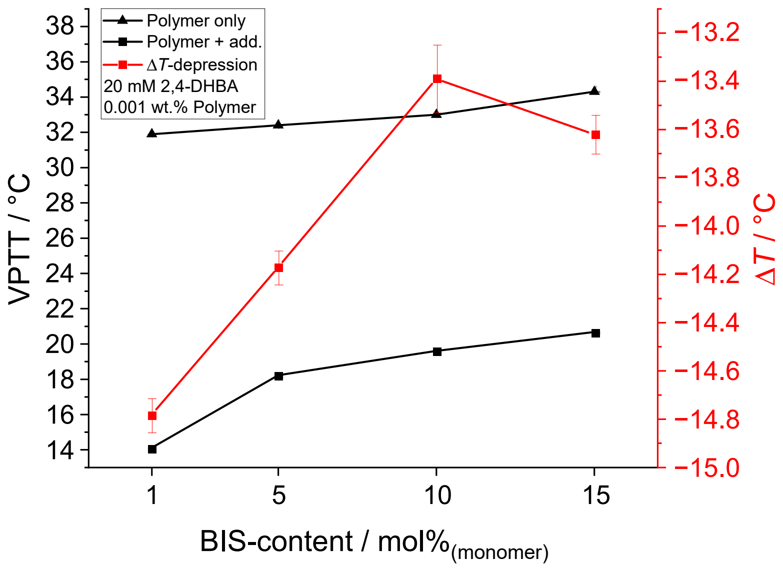

Figure S12. Plot of the VPTT (triangles: polymer only, black squares: depressed Temperature) and calculated ΔT (red squares) against crosslinker content for 2,4-DHBA.

NMR

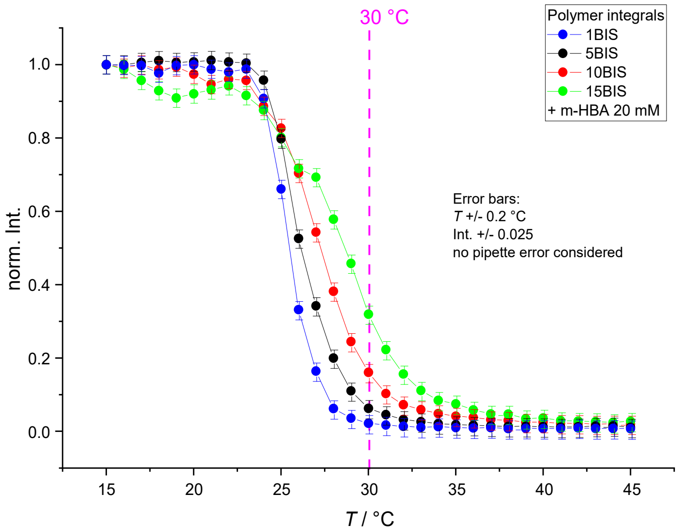

Figure S13. Integrated normalized polymer signals as acquired in ^{1}H-NMR temperature series for neat microgels containing different crosslinker content and 20 mM of *m*-HBA. Lines are guide to the eye.

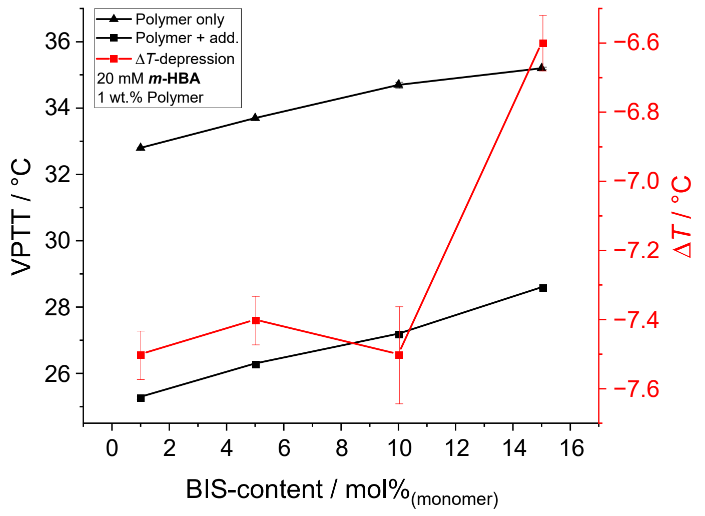

Figure S14. VPTT (triangles: polymer only, black squares: depressed Temperature) and calculated Δ*T* (red squares) against the crosslinker content for *m*-HBA.

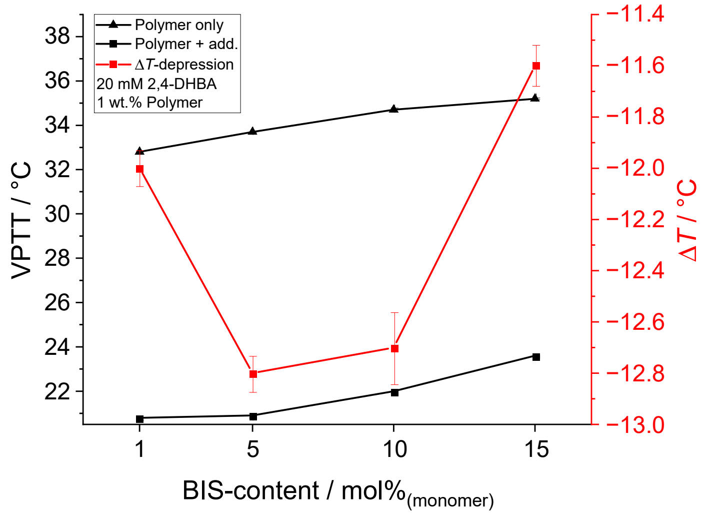

Figure S15. Plot of the VPTT (triangles: polymer only, black squares: depressed Temperature) and calculated Δ*T* (red squares) against crosslinker content for 2,4-DHBA.

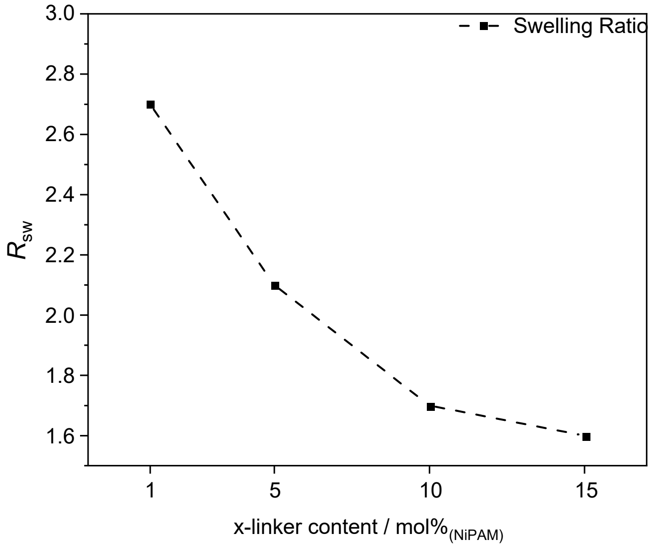

Figure S16. Swelling ratios R_{sw} for differently crosslinked microgels.
